# Effect of iodine species on biofortification of iodine in cabbage plants cultivated in hydroponic cultures

**DOI:** 10.1038/s41598-024-66575-z

**Published:** 2024-07-09

**Authors:** Péter Dobosy, Hoang Thi Phuong Nguyen, Gyula Záray, Christina Streli, Dieter Ingerle, Philipp Ziegler, Martin Radtke, Ana Guilherme Buzanich, Anett Endrédi, Ferenc Fodor

**Affiliations:** 1 Institute of Aquatic Ecology, HUN-REN Centre for Ecological Research, Karolina Út 29, 1113 Budapest, Hungary; 2 Doctoral School of Biology, ELTE Eötvös Loránd University, Pázmány Péter Sétány 1/C, 1117 Budapest, Hungary; 3 Vienna University of Technology, Atominstitut, Stadionallee 2, 1020 Vienna, Austria; 4 Bundesanstalt für Materialforschung und -prüfung, Richard-Willstätter-Straße 11, 12489 Berlin, Germany; 5 Department of Plant Physiology and Molecular Plant Biology, Institute of Biology, ELTE Eötvös Loránd University, Pázmány Péter Sétány 1/C, Budapest, 1117 Hungary

**Keywords:** Nutrition, Plant sciences

## Abstract

Iodine is an essential trace element in the human diet because it is involved in the synthesis of thyroid hormones. Iodine deficiency affects over 2.2 billion people worldwide, making it a significant challenge to find plant-based sources of iodine that meet the recommended daily intake of this trace element. In this study, cabbage plants were cultivated in a hydroponic system containing iodine at concentrations ranging from 0.01 to 1.0 mg/L in the form of potassium iodide or potassium iodate. During the experiments, plant physiological parameters, biomass production, and concentration changes of iodine and selected microelements in different plant parts were investigated. In addition, the oxidation state of the accumulated iodine in root samples was determined. Results showed that iodine addition had no effect on photosynthetic efficiency and chlorophyll content. Iodide treatment did not considerably stimulate biomass production but iodate treatment increased it at concentrations less than 0.5 mg/L. Increasing iodine concentrations in the nutrient solutions increased iodine content in all plant parts; however, the iodide treatment was 2–7 times more efficient than the iodate treatment. It was concluded, that iodide addition was more favourable on the target element accumulation, however, it should be highlighted that application of this chemical form in nutrient solution decreased the concetrations of selected micoelement concentration comparing with the control plants. It was established that iodate was reduced to iodide during its uptake in cabbage roots, which means that independently from the oxidation number of iodine (+ 5, − 1) applied in the nutrient solutions, the reduced form of target element was transported to the aerial and edible tissues.

## Introduction

Iodine (I) is an essential trace element for the human diet that is involved in the regulation of thyroid hormones. The recommended daily intake varies according to age: young children (1–8 years)—90 µg, older children (9–13 years)—120 µg, adults—150 µg, and pregnant and lactating women—220–270 µg. An insufficient amount of this element could result in iodine deficiency disorders (IDDs), such as goiter, developmental delays, and psychomotor defects, which affect around 2 billion people globally^1–3^. The use of iodized salt is a widely used strategy for eliminating IDDs; however, the iodine content of the salt may be reduced during the production steps (manufacturing, packaging, transporting), as well as cooking or frying^4,5^. Moreover, because the EU regulation has been adopted in many countries focusing on the reduction of salt consumption for the prevention of cardiovascular diseases^6^, other iodine enrichment approaches must be considered.

Iodine biofortification of fruits and vegetables appears to be a promising technique to supplement iodine in the edible parts through the use of several agronomic technologies (hydroponics, irrigation, foliar spray, fertilizer)^7–12^. Among the listed technologies, hydroponic systems as soilless cultures offer several advantages: (1) plant cultivation needs a smaller surface area, which increases yield, (2) fruits and vegetables can be grown in non-arable areas, (3) increased contact of plant roots with cultivating solutions results in favorable nutrient regulation, (4) disinfection of the solutions is easier due to the absence of microbial activity^13,14^.

It has been widely established that leafy vegetables (cabbage, Chinese cabbage, lettuce) are more suited target plants for iodine biofortification since the accumulation and transportation of this element is more efficient in these vegetables as compared to crops like fruits or fruit/root-bearing vegetables. Several studies have demonstrated that iodine is mostly transported by xylemic pathways, with concentrations decreasing from roots to the upper parts; however, phloemic transportation has also been documented in tomato and lettuce plants^15,16^. Our target plant cabbage (*Brassica oleracea*) is one of the world’s most cultivated plants, with 55 million tons grown on 2.5 million hectares in 2018 (UN Food and Agriculture Organization, 2018). Cabbage is an important vegetable with strong nutraceutical properties; for instance, consuming 100 g of fresh leaves covers 44 and 72% of daily vitamins C and K requirements, respectively^17^. However, cabbage plants also contain metabolites such as glucosinolates and their derivatives which might be responsible for negatively influencing thyroid function by inhibiting the sodium/iodide symporter in the basolateral membrane of the thyroid cells and thyroid peroxidase activity^18^. Nevertheless, recent studies imply that the previos assumptions may only be valid when raw vegetables are eaten in high amount but when cooked and consumed with proper iodine intake it is safe. For this reason, the biofortification of cabbage with optimal concentration and form of iodine would be highly beneficial.

Several studies have investigated iodine biofortification of leafy vegetables, such as cabbage, Chinese cabbage, lettuce, spinach, and celery; however, iodine supplementation studies in cabbage plants have mostly focused on growing plants in different soils and applying irrigation or fertilizer strategies. The application of iodine-containing fertilizer had diverse effects on the biomass production of cabbage. At concentration of 0.59 kg I/ha (KI), the yield was reduced^19^, but at 1–5 mg I/kg (KI or KIO_3_), it remained practically unchanged^20^. Further, plants grown in sand soil and irrigated with water containing 0.5 mg I/L (KI) showed a significant I increment in biomass^21^ as compared to the control samples. A long-term experiment was carried out by applying 5–15 kg I/ha dosage and the target element concentration amounted to 110 mg/^22^, while using 0.59 kg I/ha, 150 mg I/kg and 5.0 mg I/kg treatment dosages 12 mg/kg^20^, 1.1 mg/kg^19^ and 35 mg/kg^23^ accumulations were recorded, respectively. In our previous study, cabbage plants were irrigated with water containing 0.5 mg/L iodine, which resulted in a 10 mg I/kg concentration in the edible parts^21^.

In biofortification experiments, the efficiency of the uptake depends strongly on the physical chemical properties of the iodine containing compound, plant species, and cultivation technology. The selected iodine containing chemicals should be fulfil more requirements: high solubility in water at room temperature, high stability in solution, noncomplex forming capacity, oxidation state of iodine, low prices. Considering these requirements and successful application during biofortification experiments with leafy vegetables, potassium iodide and potassium iodate were selected as iodine sources. Some papers reported that the accumulation of iodine in soil-cultivated sweetcorn^24^ and tomato^25^ was more favorable with the application of organic iodine species; however, in the case of lettuce^11^ and spinach^26^ plants cultivated in hydroponic solution and with the addition of inorganic forms, higher accumulations could be achieved.

According to the publications referenced above, iodine uptake of cabbage plants applying different chemical forms of iodine (I^−^ or IO_3_^−^) was researched. However, in these studies the valence state of the accumulated target element was never investigated, and only the efficiency of iodine accumulation was followed.

In order to identify an effective plant-based source of iodine for nutrition of humans, the iodine accumulation of cabbage plants cultivated in hydroponic culture with iodine at concentrations ranging from 0.01 to 1.0 mg/L supplied as iodide or iodate was investigated. During the experiment, plant physiological parameters (chlorophyll content, photosynthetic efficiency), yield, and concentration changes of iodine, as well as essential elements, were monitored, and the valence state of the accumulated iodine was also determined.

Our hypotheses are the following (1) addition of iodine to the nutrient solution will not have any negative effect on the plant physiological properties in the applied concentration (2) it is expected that incrasing iodine concetration in the nutrient solution the target element accumulation will be stimulated in a considerable way (3) applying different iodine species will have various effect on the biomass production, target- and essential element transport in the different plant tissues (4) valance state of the accumulated iodine in cabbage plants has never been tested, therefore it is a key question to determnine not only the total target element concentration but its oxidation number within the plants.

## Results

### Photosynthetic efficiency and chlorophyll content in leaves

The photosynthetic efficiency and chlorophyll content of cabbage leaves are presented in Table 1. Iodine supplementation had no significant effect on the photosynthetic efficiency (p > 0.11, linear regression); it remained practically unchanged regardless of the iodine species used. The chlorophyll content of cabbage plants treated with I^−^ ranged between 42.6 and 49.6 (SPAD index), and showed a slight decrement. The presence of IO_3_^−^ in the nutrient solution resulted in moderate stimulation (48.6–52.3), but neither treatment had a significant impact compared to their controls (p > 0.055, linear regression).

**Table 1:** Chlorophyll content (SPAD index) and maximal quantum efficiency of PSII reaction centers (*Fv/Fm*) of cabbage plants cultivated in KI and KIO_3_ containing solution.

Iodine concentration in nutrient solution (mg/L)	*F*_*v*_*/F*_*m*_		SPAD index	
	I^−^	IO_3_^−^	I^−^	IO_3_^−^
Control	0.779 (2)	0.791 (1)	46.9 (11)	48.6 (9)
0.01	0.774 (3)	0.789 (2)	46.3 (9)	52.3 (13)
0.05	0.782 (1)	0.791 (1)	44.8 (10)	49.1 (15)
0.1	0.791 (2)	0.786 (1)	43.0 (9)	52.3 (11)
0.5	0.784 (1)	0.782 (2)	43.8 (12)	50.5 (14)
1.0	0.783 (1)	0.786 (2)	44.4 (11)	50.1 (14)

Data are presented as mean (n = 5), RSD% values are indicated in brackets. According to the linear regression models, none of the treated groups showed significant differences from their controls.

### Biomass production and water content of plants

**Figure 1:**
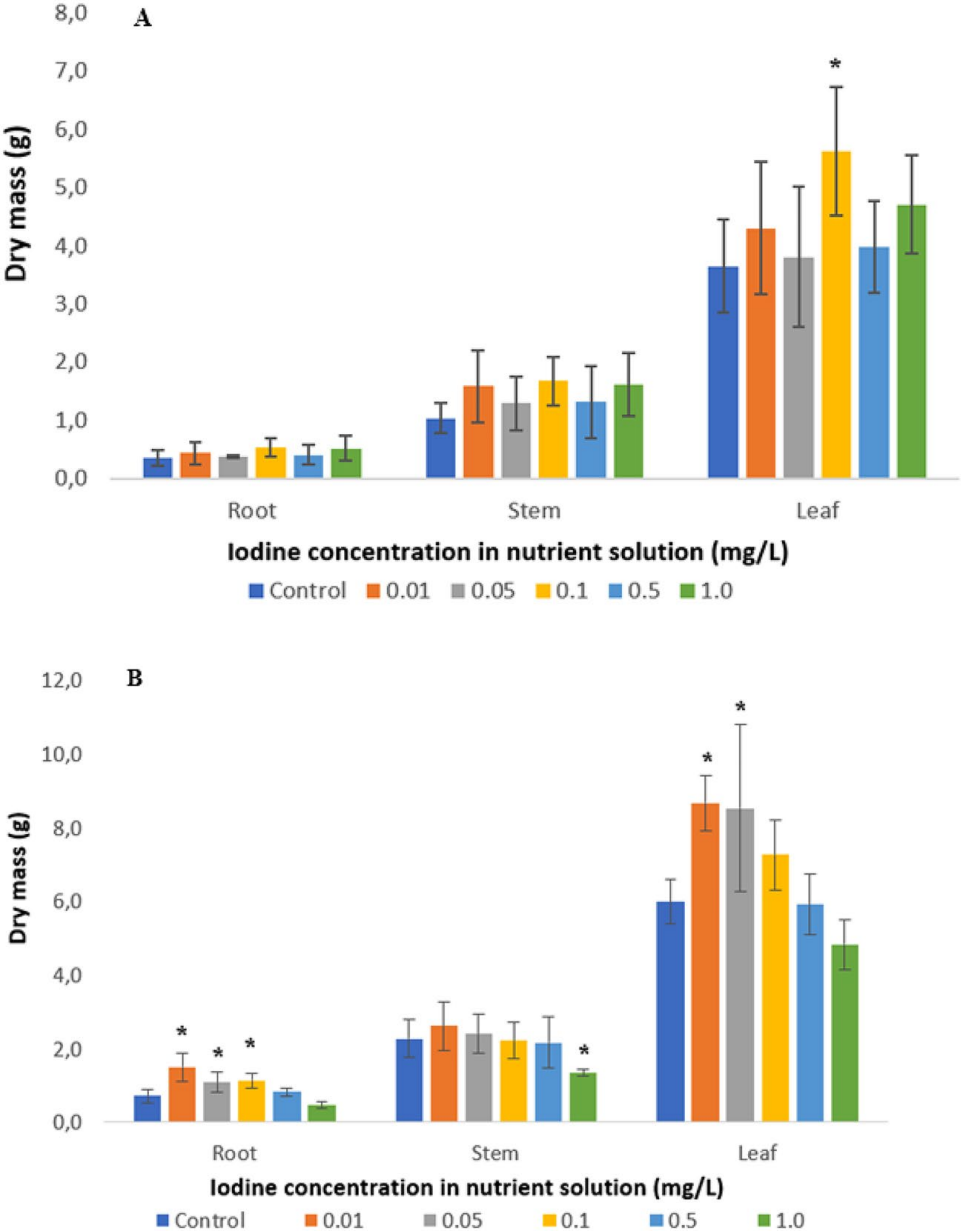
Mean dry mass of roots, stems, and leaves of cabbages cultivated in nutrient solutions containing KI (**A**) or KIO_3_ (**B**) species. Data are presented as mean (n = 5) with error bars representing RSD%. *Significant (p < 0.05) differences from the control (linear regression).

Dry masses and water contents of different cabbage plant parts treated with KI and KIO_3_ are presented in Fig. 1 and Table 2, respectively. Generally, it can be established that the addition of I^−^ had a positive effect on the yield at all dosages, although the differences were not statistically significant. The addition of IO_3_^−^ in lower concentrations (0.01 and 0.05 mg I/L) considerably increased the biomass production of roots and leaves (*p* < 0.041), while higher target element addition had no effect (p > 0.054). Applying I^−^ in the nutrient solution increased the biomass production of root, stem, and leaf samples by 16–51, 24–61, and 4–54%, respectively, relative to controls. IO_3_^−^ addition had a positive effect on the dry mass, but only at lower dosages (0.01 and 0.1 mg/L), with values increasing in root, stem, and leaf parts by 52–109, 5–15, and 21–45%, respectively. The application of 1.0 mg I/L as IO_3_^−^ exhibited a negative biomass production (19–41%, p = 0.007) compared to the control samples.

**Table 2:** Water content (g H_2_O/g dry mass) of plant parts of cabbages cultivated in KI or KIO_3_ containing nutrient solutions.

Iodine concentration in nutrient solution (mg/L)	I^−^			IO_3_^−^		
	Root	Stem	Leaf	Root	Stem	Leaf
Control	19.6 (21)	14.3 (9)	11.0 (6)	13.8 (4)	11.1 (12)	10.7 (18)
0.01	19.6 (13)	13.7 (17)	9.9 (7)	13.4 (6)	9.8 (8)	10.0 (6)
0.05	20.4 (32)	13.3 (28)	10.7 (15)	14.4 (9)	9.7 (14)	9.2 (10)*
0.1	24.3 (18)	12.6 (16)	10.2 (8)	17.1 (34)	10.0 (7)	10.7 (11)
0.5	19.5 (22)	12.4 (27)	9.2 (9)*	14.2 (8)	10.6 (12)	11.1 (7)
1.0	17.8 (19)	11.3 (4)*	8.5 (10)*	19.1 (28)*	11.9 (8)	12.3 (10)*

Data are presented as mean (n = 5), RSD% values are indicated in brackets. * indicates significant (p < 0.05) differences from the control (linear regression).

I^−^ and IO_3_^−^ had varied effects on the water content of plants. In the roots, 0.05 and 0.1 mg/L I^−^ increased the water content, whereas the highest dosage decreased it. In stems and leaves, increasing I^−^ concentration caused a continuous decrease in tissue water content. The highest I^−^ dosage caused a 9, 21, and 23% decrease in the water content of roots, stems, and leaves, respectively, compared to the control. The application of IO_3_^−^ resulted in a transient decline of water content at 0.01 mg/L in the roots, 0.01–0.5 mg/L in the stems, and 0.01–0.05 mg/L in the leaves, compared to the control. However, an increasing trend was detected at 0.05–1.0 mg/L in the roots, 1.0 mg/L in the stems, and 0.5–1.0 mg/L in the leaves, resulting in a 3, 7, and 15% increase, respectively, compared to the control.

### Iodine concentration of different plant parts

**Figure 2:**
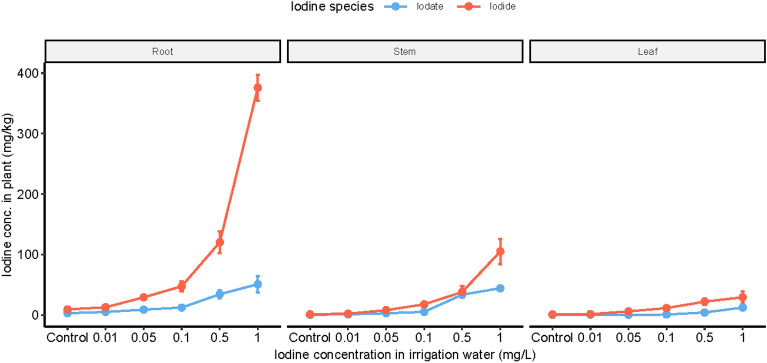
Iodine concentration in plant parts of cabbages cultivated in nutrient solutions containing KI or KIO_3_ species. Data are presented as mean and error bars represent RSD%. According to the linear regressions fitted on the log-transformed data, all treatments significantly increased the iodine concentration of all plant parts compared to the controls (p < 0.03).

Iodine concentrations in different parts of cabbage plants cultivated in KI and KIO_3_ containing nutrient solutions are presented in Fig. 2. Rising iodine dosage in the nutrient solutions resulted in significantly higher (roots and stems: p < 0.001; leaves: p < 0.03) iodine accumulation in all plant parts, with the highest iodine concentrations being observed in the roots and the lowest in the edible organs. However, the accumulation of the target element in all plant tissues varied depending on the iodine treatment type. Iodide addition showed a faster increment tendency when the treatment dosage was changed from 0.01 to 0.05 mg/L. A similar pattern was observed when iodate was added to the nutrient solution, but in this case, target element supplementation was one order of magnitude higher. In the edible tissues, the application of iodate at a concentration of 1.0 mg/L showed that with further dosage increment, more accumulation could be expected, while in the case of iodide treatment, the iodine accumulation began to be saturated. Depending on the plant tissue, the presence of iodide in the nutrient solution resulted in 2–7 times higher accumulation, compared with the iodate treatment. The application of 1.0 mg I^−^/L resulted in the accumulation of 376, 105, and 29.2 mg/kg iodine in the root, stem, and leaf (DW), while with the iodate treatment, the accumulation was 50.9, 42.2, and 12.2 mg/kg, respectively. Based on the iodine concentration and fresh weight of edible plant tissues, it can be calculated that consuming 32.3 g (KI) or 77.0 g (KIO_3_) would cover the daily iodine need (180 µg) of a normal adult. On the basis of the dry mass and target element accumulation in the tissues, the iodine distribution among the different plant parts is depicted in Table 3.

**Table 3:** Iodine distribution percentage (%) among the plant parts of cabbages cultivated in KI or KIO_3_ containing nutrient solutions.

Iodine concentration in nutrient solution (mg/L)	I^−^			IO_3_^−^		
	Root	Stem	Leaf	Root	Stem	Leaf
Control	48	14	39	49	15	36
0.01	42	21	36	43	33	24
0.05	23	30	47	47	35	18
0.1	29	33	38	45	37	18
0.5	26	27	47	23	53	24
1.0	39	34	27	17	42	41

Comparing the two treatment types in control samples, the iodine distribution displayed a similar trend in the root, stem, and leaf parts resulting in 48–49, 14–15, and 36–36%, respectively. The application of iodine, independent of its chemical form, shifted the ratio to the upper parts (stem + leaf), with the distribution changing from 52 to 58–77% in the case of I^−^ treatment, and from 51% to 53–83% in the case of IO_3_^−^. Based on the ratio of average iodine concentration in the leaf and root tissues, translocation factors (TF) were calculated and the results are listed in Table 4.

**Table 4:** Translocation factor of iodine in cabbage plants cultivated in KI or KIO_3_ containing nutrient solutions.

Iodine concentration in nutrient solution (mg/L)	I^−^	IO_3_^−^
Control	0.08	0.09
0.01	0.09	0.10
0.05	0.22	0.05
0.1	0.13	0.06
0.5	0.18	0.14
1.0	0.08	0.24

In the control samples, the TF was 0.08–0.09. The application of iodine in the nutrient solution had a positive influence on the TF, but at the highest dosage, the TF was reduced relative to the control value. In contrast, IO_3_^−^ treatment at low concentrations (0.01–0.1 mg/L) resulted in a decrease in plant TF values, but higher dosages (0.5 and 1.0 mg/L) resulted in an increase.

### Valence state of iodine in cabbage roots

**Figure 3:**
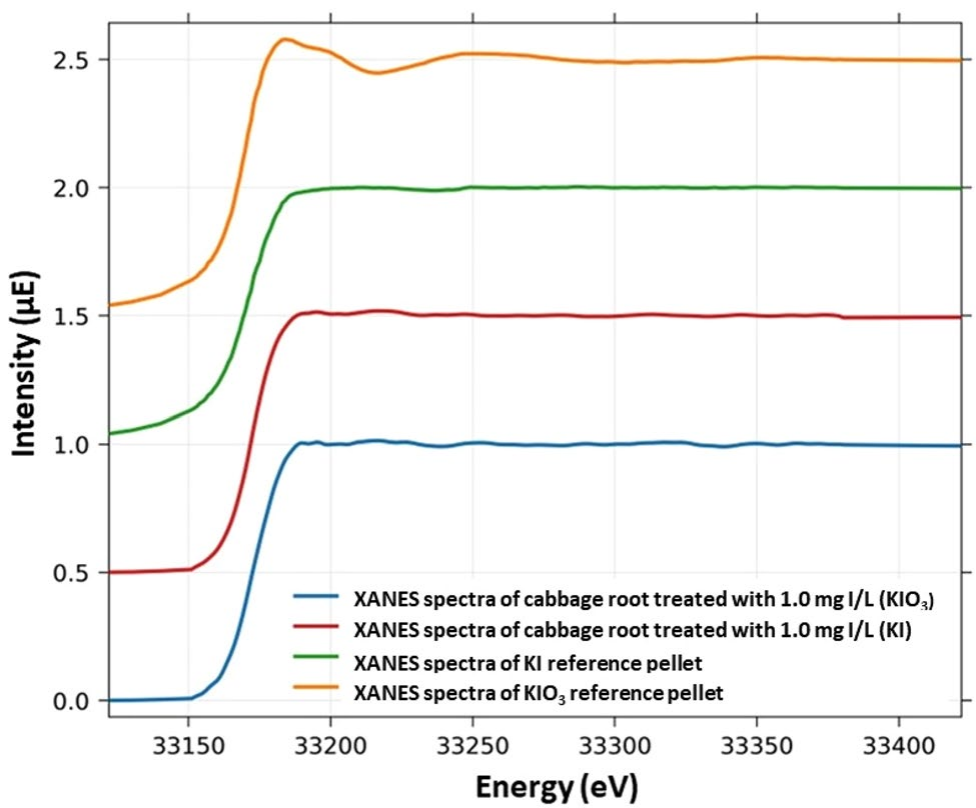
I-K edge XANES spectra of KI and KIO_3_ reference materials and cabbage roots cultivated in nutrient solutions containing KI or KIO_3_ species.

For the characterization of the valence state of iodine in cabbage roots, the XANES spectra of KI and KIO_3_ reference materials, as well as root samples treated with 1.0 mg/L iodide, are presented in Fig. 3*.*

The XANES spectra of root tissues of plants cultivated in KI or KIO_3_-containing nutrient solution were found to be very similar to that of the KI reference material rather than the KIO_3_ standard. In both treatment types, the accumulated iodine form in the roots was I^−^. IO_3_^−^ was converted to I^−^, indicating that reductive chemical reactions are dominant in the roots.

### Essential elements transport in the plant parts

Average concentrations of selected macro- (Ca, Mg, P, K) and micro-nutrients (B, Mn, Cu, Zn, Fe) in the different tissues of plants cultivated in nutrient solutions with various iodine concentrations are demonstrated in Tables 5 and 6. I^−^ treatment at 0.05–0.5 mg/L dosage had a positive effect on Ca, P, Mg, B, and Fe concentration of root samples, and when the highest treatment was applied, C and Zn content was moderately increased compared to the control samples. An inhibitory effect was observed in stems for Ca, P, and Zn in all treatments, and using 1.0 mg I^−^/L in the nutrient solution, the concentration of all elements was reduced. Iodide addition had a negative effect on the transport of essential elements, with concentrations of the selected macro- and micro-elements decreasing by 2–50%. The application of 0.01–0.5 mg/L IO_3_^−^ had a negative effect on Zn, Fe, B, and Ca concentrations in all treatment steps in the roots. In stem tissues, 0.01–1.0 mg IO_3_^−^/L stimulated the B content, while the 0.01–0.05 mg/L dosage inhibited the accumulation of the other six elements, compared to the control samples. Similarly, the B concentration of leaf samples was increased in all iodate dosages (2–48%). Applying 0.05 mg/L iodate in the nutrient solution hindered the accumulation of all essential elements, while with application of 1.0 mg/L iodate enhanced the concentrations. Comparing the application of the two iodine species in the nutrient solution and focusing on the edible part of the cabbage plant, it can be summarized that the iodate treatment had a less negative influence on the nutrient transport of the leaves. The differences are especially conspicuous in the case of Zn, Fe, and K, where the addition of IO_3_^−^ and I^−^ resulted in concentration changes of + 48, + 42, + 36% and − 49%, − 47%, − 43%, respectively, relative to the controls.

**Table 5:** Concentrations of selected micro- and macronutrients in plant parts of cabbages cultivated in control and KI containing nutrient solutions.

Element	Plant part	Iodine concentration in nutrient solution (mg/L)					
		Control	0.01	0.05	0.1	0.5	1.0
Ca (mg/kg)	Root	3021 (24)	8040 (27)*	7195 (15)*	6181 (22)*	4935 (37)*	3018 (20)
	Stem	16,514 (18)	14,507 (24)	15,292 (19)	15,231 (24)	13,496 (21)	11,355 (24)*
	Leaf	53,817 (19)	45,670 (26)	50,926 (13)	40,253 (11)*	35,019 (12)*	34,733 (12)*
Mg (mg/kg)	Root	2182 (12)	2104 (23)	2399 (17)	2445 (17)	2324 (17)	2279 (6)
	Stem	3830 (28)	3910 (18)	3835 (14)	4680 (18)	3833 (32)	2555 (30)*
	Leaf	8905 (14)	7461 (13)*	8174 (14)	7377 (20)*	6089 (11)*	6381 (16)*
K (mg/kg)	Root	71,825 (15)	71,274 (17)	89,997 (23)	60,203 (20)	72,660 (27)	40,427 (14)*
	Stem	109,135 (22)	87,838 (29)	101,058 (23)	85,271 (25)*	69,585 (27)*	62,552 (9)*
	Leaf	57,013 (15)	46,719 (29)	46,168 (11)	38,935 (22)*	33,793 (26)*	30,028 (20)*
P (mg/kg)	Root	12,224 (30)	15,056 (19)*	17,892 (18)*	14,060 (14)	14,839 (7)	10,967 (11)
	Stem	4867 (13)	4523 (17)	4546 (6)	4339 (12)	4266 (9)	4165 (16)
	Leaf	5701 (15)	4672 (29)	5198 (7)	5292 (23)	4393 (11)*	4262 (18)*
B (mg/kg)	Root	41.7 (30)	60.8 (17)*	80.8 (17)*	62.1 (19)*	73.3 (18)*	31.5 (17)*
	Stem	35.4 (19)	30.1 (9)	35.3 (16)	35.9 (13)	28.3 (15)*	27.6 (12)*
	Leaf	68.1 (3)	66.9 (23)	63.7 (32)	68.5 (11)	55.0 (25)*	59.0 (33)
Mn (mg/kg)	Root	1572 (27)	1326 (29)	2044 (16)	1200 (23)	1481 (13)	649 (20)*
	Stem	36.6 (23)	28.0 (19)	38.6 (30)	33.3 (14)	28.9 (7)	26.5 (11)*
	Leaf	343 (22)	288 (19)	329 (22)	261 (18)*	216 (20)*	231 (21)*
Cu (mg/kg)	Root	61.5 (28)	52.4 (12)	77.7 (20)	50.1 (20)	66.9 (18)	61.8 (17)
	Stem	2.20 (27)	1.72 (20)	2.87 (26)	2.17 (27)	1.78 (19)	1.38 (16)*
	Leaf	6.1 (27)	5.3 (28)	5.6 (17)	4.0 (19)*	3.0 (20)*	3.1 (27)*
Zn (mg/kg)	Root	117 (10)	132 (20)	121 (12)	110 (20)	136 (27)	127 (17)
	Stem	16.9 (12)	10.2 (5)*	16.0 (14)	14.3 (18)	15.2 (22)	12.4 (20)*
	Leaf	47.4 (19)	41.5 (26)	43.5 (16)	37.0 (9)	39.7 (32)	38.3 (10)
Fe (mg/kg)	Root	5344 (21)	6271 (7)	7106 (17)*	6216 (20)	6741 (33)	6330 (17)
	Stem	7.27 (14)	3.77 (23)	16.8 (20)*	11.7 (22)*	16.4 (16)*	5.70 (20)
	Leaf	69.0 (15)	48.6 (10)*	51.1 (10)*	45.7 (17)*	43.9 (26)*	39.2 (20)*

Data are presented as mean (n = 5), RSD% values are indicated in brackets. * indicates significant (p < 0.05) differences from the control (linear regression).

**Table 6:** Concentrations of selected micro- and macronutrients in plant parts of cabbages cultivated in control and KIO_3_ containing nutrient solutions.

Element	Plant part	Iodine concentration in nutrient solution (mg/L)					
		Control	0.01	0.05	0.1	0.5	1.0
Ca (mg/kg)	Root	1254 (19)	1131 (27)	810 (36)*	686 (34)*	661 (22)*	590 (34)*
	Stem	12,613 (29)	6688 (15)*	7751 (22)*	8386 (20)*	9155 (22)*	8649 (15)*
	Leaf	43,004 (3)	40,285 (11)	37,040 (9)	39,874 (20)	45,308 (9)	50,438 (5)
Mg (mg/kg)	Root	1890 (16)	1949 (9)	2058 (8)	2196 (18)	2223 (26)	1436 (25)
	Stem	5122 (19)	4265 (16)	4673 (23)	5279 (16)	5155 (9)	4831 (16)
	Leaf	8260 (17)	8683 (12)	7748 (20)	8404 (8)	9066 (8)	9419 (7)
P (mg/kg)	Root	7991 (14)	8526 (28)	8008 (19)	7970 (14)	8427 (21)	9728 (13)
	Stem	7019 (23)	6011 (8)	6854 (20)	7001 (9)	6682 (7)	6482 (14)
	Leaf	5267 (27)	4672 (12)	4918 (22)	5313 (15)	5854 (23)	6054 (6)
K (mg/kg)	Root	23,724 (20)	31,083 (19)	29,521 (21)	29,783 (32)	22,856 (34)	18,089 (3)
	Stem	57,601 (14)	41,825 (7)*	50,112 (22)	54,294 (10)	58,177 (13)	61,856 (17)
	Leaf	26,946 (12)	23,014 (14)	26,228 (28)	26,121 (17)	30,444 (21)	36,713 (6)*
B (mg/kg)	Root	25.5 (10)	24.2 (12)	22.1 (10)	21.4 (10)	21.6 (8)	23.6 (13)
	Stem	32.8 (9)	34.2 (9)	36.5 (14)	40.4 (10)*	38.2 (11)	39.9 (9)*
	Leaf	69.8 (15)	79.4 (17)	73.4 (21)	77.3 (8)	81.5 (17)	71.0 (6)
Mn (mg/kg)	Root	341 (26)	290 (26)	433 (22)	306 (18)	393 (36)	708 (15)*
	Stem	50.1 (20)	31.8 (17)*	35.1 (26)*	39.0 (20)*	46.2 (8)	54.6 (16)
	Leaf	349 (10)	302 (8)	273 (30)*	318 (17)	366 (10)	413 (5)
Cu (mg/kg)	Root	24.8 (16)	19.3 (31)*	28.2 (21)	21.1 (11)	21.6 (9)	27.8 (26)
	Stem	2.33 (16)	1.76 (11)*	2.07 (22)*	2.43 (21)	2.47 (18)	2.88 (19)*
	Leaf	2.80 (22)	1.62 (26)	1.43 (21)	2.88 (17)	3.15 (23)	4.15 (24)
Zn (mg/kg)	Root	33.6 (15)	28.6 (18)	31.6 (5)	28.2 (13)	32.7 (23)	33.5 (11)
	Stem	18.7 (19)	18.4 (28)	15.8 (23)	19.9 (19)	16.4 (21)	16.3 (20)
	Leaf	34.3 (12)	25.4 (8)*	28.3 (27)	26.8 (28)*	32.6 (10)	42.9 (21)
Fe (mg/kg)	Root	4483 (13)	3178 (15)*	3387 (19)*	3230 (14)*	4014 (20)	5937 (28)
	Stem	16.8 (34)	14.0 (29)	10.5 (27)*	19.3 (21)	12.7 (30)	22.1 (33)
	Leaf	37.8 (22)	15.8 (9)*	22.7 (12)*	30.2 (28)	35.6 (19)	53.5 (27)

Data are presented as mean (n = 5), RSD% values are indicated in brackets. * indicates significant (p < 0.05) differences from the control (linear regression).

## Discussion

In this study, the effect of iodine (in different chemical forms) supplemented nutrient solution on plant physiological properties, biomass production, and uptake and translocation of iodine and essential elements in cabbage plants were investigated in addition to the determination of valence state of the accumulated iodine in the roots.

Iodine addition had no influence on the photosynthetic efficiency, and in the presence of IO_3_^−^ only a modest increase in chlorophyll content was observed. Only a few studies have focused on these parameters in plants treated with iodine. Iodine uptake and translocation were investigated in cabbage plants after irrigation with iodide-containing water at concentrations 0.1–0.5 mg/L, and Chl* a* and *F*_*v*_*/F*_*m*_ values remained nearly unaltered^21^. Iodine biofortification of cabbage was studied using fertilizers containing 5–15 kg I/ha in I^−^ and IO_3_^−^ forms, and no photosynthetic effects (necrosis, defoliation, and chlorosis) were observed^22^. In lettuce plants cultivated in hydroponic solution containing 2.5–10 mg I/L (I^−^ or IO_3_^−^), iodate treatment stimulated the chlorophyll content in the leaf tissues while iodide had no effect when compared to the control samples^27^, which is consistent with our findings. The photosynthetic efficiency of buckwheat microgreens immersed in water containing 1000 mg I/L (I^−^ or IO_3_^−^) was 0.78–0.80, similar to cabbage plants, indicating that iodine addition, even at relatively high concentrations, had no influence on this parameter^28^. The effect of iodine on plant physiological parameters is evident, with iodate demonstrating a positive effect and iodide having no effect.

Only a few papers deal with the iodine biofortification of cabbage and yield variations, and in most cases, the plants were cultivated in soil with iodine supplementation through irrigation, fertilizer, or foliar spray techniques. In cabbage plants grown in three soil types with varying physico-chemical properties and irrigated with iodine (KI)-containing water at concentrations 0.1–0.5 mg/L, biomass production did not differ significantly from controls^21^. The use of 0.59 kg I/ha as KI solid fertilizer lowered the growth of cabbage by 46%^19^. In Chinese cabbage plants cultivated in soilless culture containing 0.1–5.0 mg I/L (I^−^ or IO_3_^−^)^29^ and growing in soils treated with 10–150 mg KI/kg^30^, the biomass production was inhibited with all treatment types. Our results showed that iodate treatment had no effect on the yield and that iodide addition to the nutrient solution stimulated the biomass production of the cabbage plants, which contradicts the observations of the cited papers. However, it should be noted that in hydroponic systems, elements (e.g., iodine) have more direct contact with the roots, improving iodine compounds absorption and resulting in a variety of effects. Iodate treatment improved the water content of the roots and leaves, whereas iodide treatment decreased it, indicating that the plants may be more sensitive to the latter due to altered water relations. Such an effect was observed in arsenite-treated cucumber plants, where the redox transformation of arsenic resulted in oxidative stress^31^. But iodide treatment was not coupled with growth inhibition, and the relatively unchanged *F*_*v*_*/F*_*m*_ stress index also indicates healthy plant growth. Hence, the observed shift in tissue water content can only be explained by the differing effects of the two iodine forms on K accumulation, which varied in tandem with water content.

In literature, iodine biofortification studies in cabbage plants have focused mainly on applying fertilizer, spray, or irrigation technologies. Increasing the iodine concentration in the hydroponic solution resulted in higher target element accumulation when I^−^ or IO_3_^−^ supplementation was used—these observations have been documented in several other studies. On the basis of our results, applying 1.0 mg I/L iodide and iodate treatment, the iodine concentrations in edible part tissues of cabbage plants were 29.2 and 12.2 mg/kg, respectively. It suggests that iodide was more bioavailable for the target plant, with a ~ 2.5 times higher accumulation factor than iodate treatment. Comparable results were reported in Chinese cabbage with the same iodine dosage, with accumulation being about two times larger with the I^−^ treatment^29^. In contrast, treating lettuce with fertilizer containing 1–15 kg I/ha IO_3_^−^ resulted in lower target element accumulation than I^−^^32^. It was noted that when KI and KIO_3_ were applied as solid fertilizer or foliar spray at concentrations 1.0–5.0 mg/kg, at the highest iodine dosage, the accumulation of I^−^ and IO_3_^−^ was comparable^20^. In a long-term experiment (2017 and 2018), 5–15 kg I/ha KI and KIO_3_ containing fertilizer was used, and it was established that when comparing the two treatment types, iodine accumulation in the first year was not statistically different, whereas, in the next year, KI addition increased iodine uptake by 34% over iodate^22^. To summarize, the effect of the administered iodine species on target element accumulation is strongly dependent on the plant, treatment types, and dosages.

In general, soil has been used as a growth medium in research to investigate the iodine biofortification of cabbage plants. When plants were grown in three distinct soil types (sand, sandy silt, silt) and irrigated with iodine (KI) at a concentration of 0.5 mg I^−^/L, the target element concentrations in the edible part were 3.71–10.0 mg/kg, depending on the soil type^21^. A two-year-long experiment was conducted to study the iodine uptake of cabbages using KI and KIO_3_-containing fertilizers with iodine concentrations of 1.0–5.0 mg/kg, and the maximum iodine concentration obtained was 12.2 mg/kg^20^. Further, while using 0.59 and 15 kg I/ha as KI, it was 1.10 mg/kg^19^ and 110 mg/kg^22^, respectively. Two similar experiments were conducted with KI-containing fertilizer at concentrations of 10–150 mg I/m^2^, and maximum iodine concentration of 35 mg/kg was reported^23,33^. In iodine biofortification technologies targeting cabbage plants, the efficiency of iodine uptake is strongly dependent on the chemical form and concentration of applied iodine, as well as the growth medium. Considering our results, cultivating cabbage plants in hydroponic solutions containing 1.0 mg I/L (KI) indicated favourable results, with 29.2 mg I/kg detected in the edible tissues.

Iodine biofortification strategies in cabbage plants have mostly focused on concentration changes of total iodine; nevertheless, it is equally important to determine the chemical form of the accumulated element. In literature, only one paper dealing with the transport of iodine species in rice, barley, and soybean is accessible, and it reveals that IO_3_^−^ gets converted to I^−^ by the iodate reductase enzyme in the roots of the investigated plants^34^. Based on our XANES data, it was established that reductive reactions are dominant in the roots, regardless of the iodine species added to the nutrient solutions. At the end of the experiment, iodide was the predominant chemical form, suggesting that cabbage roots have the same iodate reduction activity as rice, barley, and soybean. A similar experiment was conducted to determine the arsenic species in cucumber xylem sap by cultivating plants in 0.15 mg/L arsenic (arsenite and arsenate) containing nutrient solution, and, in the highest concentration, arsenite occurred in the sap samples^35^.

Although several studies have explored the changes in biomass production and iodine uptake in cabbage plants, a limited number of papers have detailed the effect of iodine supplementation on the transport of essential elements. In our former study, cabbage plants were cultivated in different soil types and irrigated with iodine-containing water (0.5 mg I^−^/L), and the treatment decreased B and Fe while maintaining P, Zn, and Cu concentrations in cabbage leaves^21^. These observations are partly in line with our present experimental data, where the application of iodide at 0.05–1.0 mg I^−^/L dosages hampered the transport of all essential elements to the leaves. The iodate treatment, on the other hand, increased the transport of essential nutrients to the leaves. As iodate has a higher translocation factor at 1.0 mg I^−^/L and iodine distribution within the plant also shows the more efficient translocation of iodine from iodate source, the iodine translocation to the leaves is positively correlated with the translocation of other elements. XANES evidences iodate reduction to iodide in the roots but it is unclear how the redox transformation may impact the translocation of iodine and other essential elements.

## Conclusions

Our study shows that not only iodine accumulation can be achieved but also essential element concentrations can be increased with the right choice of iodine form and dosage. Neither iodide nor iodate in the concetration range of 0.1–1.0 mg I/L caused considerable change in physiological parameters or the dry mass of the plants compared to the control but iodide showed higher accumulation potential in the leaves than iodate. It was concluded that applying 1.0 mg I/L iodide in the nutrient solution resulted in the best target element accumulation, however, iodate increased while iodide decreased the essential element concentrations in the leaves in general. As iodate reduction to iodide was detected in the roots and most possibly the translocated form remained iodide, the specific site and mechanism of the reduction and the subsequent metabolic changes leading to increased transport of other elements remain to be elucidated.

As iodine biofortifiacation of cabbage plants cultivated in hydroponic system were not or poorly investigated, and chemical species of the accumulated target element has not been identified before, our experimental results could contribute to improving iodine biofortification strategies in the agronomy with new aspects.

## Material and methods

### Plant cultivation in hydroponic culture

Cabbage (*Brassica oleracea* cv. Szentesi lapos) seeds were germinated on filter paper moistened with deionized water in Petri dishes under diffuse sunlight at room temperature for 7 days. Seedlings of the same size (about 3 cm hypocotyl) were selected and rolled up in a sponge strip which was then placed in a circular polystyrene plate (d = 13 cm) with a hole in the center (d = 35 mm). The plates were then inserted in a pot filled with 2 dm^3^ modified quarter-strength Hoagland nutrient solution (Table 7).

**Table 7:** Composition of modified Hoagland solution.

Macronutrient	Concentration (mM)	Micronutrient	Concentration (µM)
KNO_3_	1.25	Fe(III)-citrate-hydrate	25.0
Ca(NO_3_)_2_	1.25	MnCl_2_⋅4H_2_O	4.5
MgSO_4_	0.50	ZnSO_4_⋅7H_2_O	0.19
KH_2_PO_4_	0.25	Na_2_MoO_4_⋅2H_2_O	0.12
		CuSO_4_⋅5H_2_O	0.08
		H_3_BO_3_	11.6

The floating plates were covered with black nylon foil and the nutrient solution was continually aerated and changed once a week with fresh solution. After three weeks, iodine at concentrations of 0.01, 0.05, 0.1, 0.5, and 1.0 mg/L as KI or KIO_3_ was added to the nutrient solutions. Each treatment group consisted of five different plants grown in separate pots. The plants were grown in a climate-controlled growth chamber (located in the Faculty of Science, ELTE Eötvös Loránd University, Budapest) at 20/25 °C, 60% relative humidity, and 200 µmol/m^2^/s photosynthetic photon flux density (PPFD) with a 10/14 h dark/light period. After one month of iodine treatment, the plants were harvested. The whole experiment was performed twice.

#### Chlorophyll content and photosynthetic efficiency

Total chlorophyll content was determined before the harvest using a SPAD 502+ portable chlorophyll meter (Konica-Minolta, Osaka, Japan) on the two youngest fully developed leaves. Chlorophyll *a* fluorescence induction measurement was conducted with leaf discs using a PAM 101–102–103 Chlorophyll Fluorometer (Walz, Effeltrich, Germany). Plant leaves were sampled with a cork borer (d = 5 mm), and discs moistened with deionized water were dark-adapted for 20 min in a sample holder. Determination of the *F*_*0*_ level of fluorescence was carried out by turning on the measuring light (modulation frequency of 1.6 kHz and PPFD less than 1 μmol/m^2^/s) after 3 s of illumination with far-red light to eliminate reduced electron carriers^36^. The maximum fluorescence yield of the dark-adapted stage, *F*_*m*_, was measured by exerting a 0.7 s pulse of white light (PPFD of 3500 μmol/m^2^/s, light source: KL 1500 electronic, Schott, Mainz, Germany). The maximal quantum efficiency of the PSII reaction center (*F*_*v*_*/F*_*m*_) was calculated as:

1 $${F}_{v}/{F}_{m}=\left({F}_{m}-{F}_{0}\right)/{F}_{m}$$

#### Determination of fresh and dry weights

At the end of the growth period, the different plant organs (root, stem, and leaf) were separated and fresh weights (FW) were measured with an analytical balance. An aliquot mass of cabbage roots was separated and kept for the determination of the valence state of iodine by the XANES technique. For the determination of dry weight (DW), the plant samples were washed with ultrapure water, dried in a laboratory oven at 40 °C for 2 days, and reweighed. The water content of the plant parts was calculated using the Eq. (3):

2 $$Water \;content=(FW-DW)/DW$$

### Chemicals

All chemicals used in the experiment were of analytical grade. For plant cultivation and preparation of standard solutions, ultrapure water was produced by a WasserLab Automatic unit (Labsystem Ltd., Budapest, Hungary). Iodine stock solutions were prepared using KI and KIO_3_ solid salts (Sigma Aldrich Ltd., Missouri, USA) and for the determination of essential macro- and microelements (Ca, Mg, K, P, B, Mn, Cu, Zn, Fe) a multi-element standard solution (Sigma Aldrich Ltd., Missouri, USA) was applied. For measurement of iodine species by X-ray absorption near-edge spectroscopy (XANES), pellet standards were prepared from a mixture of KI or KIO_3_ and cellulose powder (Sigma Aldrich Ltd., Missouri, USA). Accuracy of the elemental analyses by inductively coupled plasma mass spectrometer was verified by applying a NIST 1573a tomato leaf standard reference material (National Institute of Standards and Technology, Gaithersburg, United States).

#### Sample preparation and elemental analyses

The dried samples were homogenized with a Retsch GM 200 machine (Labsystem Ltd., Budapest, Hungary). 100–500 mg solid samples were mineralized in a mixture of 7 mL Suprapur® 65% nitric acid (VWR International, Pennsylvania, USA) and 3 mL Suprapur® 30% hydrogen-peroxide (VWR International, Pennsylvania, USA), followed by digestion of plant parts in a TopWave microwave-assisted digestion system (Analytik Jena, Jena, Germany). The solutions were transferred into metal-free plastic centrifuge tubes and filled up to 25 mL with ultrapure water. The concentrations of iodine, as well as essential macro- and micro-elements were determined by applying a PlasmaQuant MS Elite inductively coupled plasma mass spectrometer (Analytik Jena, Jena, Germany). Recovery values of iodine and essential nutrient elements (Ca, Mg, K, P, B, Mn, Cu, Zn, Fe) by analysing tomato leaf standard reference material amounted to 95–105%. The translocationfer factors (TF) for iodine were calculated as follows:

3 $$TF=I \;concentration \;in\; leaf/I \;concentration\; in\; root$$

#### Determination of iodine species

For XANES measurements, house-made standards were prepared by mixing iodine salts (KI or KIO_3_) with cellulose powder in a 1:3 ratio and pressing them into pellets with diameters and thickness of 10 and 3 mm, respectively. Dried root samples treated with the highest iodine dosage were ground and homogenized using an agate mortar and pestle. From this powdered plant material, similar pellets were prepared by applying 10 tons/cm^2^ pressure for 1 min. The valence state of iodine was determined by the XANES technique at the Beamline of BESSY (Berlin, Germany). Iodine K-edge XANES (I K-edge (33.17 keV) was applied since the L-lines of iodine (Lα: 3.93 keV) overlap with Ca (Kα: 3.6 keV). The XANES measurements were performed in fluorescence mode. The Beamline was ideal for this experiment as it is equipped with a Superconducting 7 T wavelength shifter that provides sufficient high-energy photons. A Si (111) double crystal monochromator and a 4-element Silicon Drift detector were used for measuring the fluorescence radiation of I-K radiation. The peak deconvolution was performed using PyMCA^37^, and the XANES data were evaluated using Athena-IFEFFIT^38^.

#### Statistical evaluation

R statistical software (R Core Team, 2020) was used for data analysis. The effects of treatments and iodine species on photosynthesis, dry mass, and element content of plants were tested with linear regression models (lm function of the ‘stat’ package). Separate models were built for each plant part and potential response variable (*Fv/Fm*, chlorophyll content, dry mass, water content, and all elements), with the same set of explanatory variables: treatment (as a 6-level factor), iodine species (as a 2-level factor), and their interaction. This later demonstrated how different the reactions of plants to the same concentrations of the different iodine species were. The assumption of the linear models was checked before running the models, and for some explanatory variables (e.g., water content, iodine, and other element concentrations of the plants), logarithmic transformation was required for a good model fit.

### Experimental statement

The experiment complied with relevant institutional, national, and international guidelines and legislation.

## Data Availability

Original experimental data are available from the corresponding author upon a request.
